# Repeated imaging through a multimode optical fiber using adaptive optics

**DOI:** 10.1364/BOE.448277

**Published:** 2022-01-10

**Authors:** Carla C. Schmidt, Raphaël Turcotte, Martin J. Booth, Nigel J. Emptage

**Affiliations:** 1 Department of Pharmacology, University of Oxford, Mansfield Road, Oxford OX1 3QT, United Kingdom; 2 Tech4Health Institute, NYU Langone Health, New York, NY 10010, USA; 3 Department of Engineering Science, University of Oxford, Parks Road, Oxford OX1 3PJ, United Kingdom; 4 These authors contributed equally

## Abstract

Multimode optical fibers (MMF) have shown considerable potential for minimally invasive diffraction-limited fluorescence imaging of deep brain regions owing to their small size. They also look to be suitable for imaging across long time periods, with repeated measurements performed within the same brain region, which is useful to assess the role of synapses in normal brain function and neurological disease. However, the approach is not without challenge. Prior to imaging, light propagation through a MMF must be characterized in a calibration procedure. Manual repositioning, as required for repeated imaging, renders this calibration invalid. In this study, we provide a two-step solution to the problem consisting of (1) a custom headplate enabling precise reinsertion of the MMF implant achieving low-quality focusing and (2) sensorless adaptive optics to correct translational shifts in the MMF position enabling generation of high-quality imaging foci. We show that this approach achieves fluorescence imaging after repeated removal and reinsertion of a MMF.

## 1. Introduction

Imaging subcortical brain structures in living animals is essential to understand the link between subcellular processes and higher cognitive functions such as learning and memory [1,2]. To dissect mechanisms underlying neural function and circuit plasticity, chronic imaging of synaptic structures and their dynamics is necessary. However, due to scattering and optical aberrations within brain tissue, high-resolution imaging of structures deeper than ∼1 mm over longer periods has been challenging [3]. Three-photon fluorescence microscopy and adaptive optics (AO) have, together and separately, enabled high resolution imaging of deep cortical layers and beyond. AO corrects optical aberrations, usually those imparted by the tissue, while scattering is alleviated in three-photon fluorescence microscopy by longer excitation wavelengths [4–6]. However, both technologies represent an extension of the depth ranges accessible for optical microscopy when imaging from the surface of the brain. Endoscopes, devices inserted into tissue to relay light for imaging, are still required to reach many brain regions, even in small mammals such as mice.

Micro-endoscopic approaches should ideally offer unlimited imaging depth, cause minimal damage to the surrounding tissue and disruption of the neuronal network, provide subcellular spatial resolution for synapse visualization, and be compatible with imaging applications in freely behaving animals. In addition, studying synaptic connectivity and plasticity deep within living brains over longer time-periods requires the development of systems capable of repeated imaging. Widely used endo-microscopes commonly comprise a gradient refractive index (GRIN) lens or a fiber bundle [7–10]. Deep brain imaging using GRIN lenses or fiber bundles enables subcellular resolution and is suitable for longitudinal studies [11,12]. However, while significant advances for large volume *in vivo* imaging have been achieved with methods employing similarly sized waveguide [13], the implantation of these probes necessitates the removal of cortical tissue or the use of guide cannulas, which are typically between 0.6 - 1 mm in diameter [11,14,15]. Hence, these imaging tools involve substantial damage to brain tissue, which is accompanied by inflammation and gliosis [15] and is thought to negatively affect neuronal network function [16]. In order to investigate physiological synaptic processes and plasticity, that are minimally altered by tissue damage, the development of minimally invasive endo-microscopes is desirable.

More recently, multimode optical fibers (MMF) have been used as the principal component of micro-endoscopes for diffraction-limited fluorescence imaging in deep brain regions *in vivo* [17–19]. Due to their small size, with diameters typically ∼ 100 µm, MMF have a smaller footprint than GRIN lenses and multicore fibers, and they thus generate minimal tissue damage when inserted into the brain [17,19]. Importantly, MMF-based endoscopes offer a unique capacity for *in vivo* fluorescence imaging: their small size poses little limitation in how deep and where MMF can be inserted. Indeed, MMF can reach any region in a mouse brain. Moreover, given the considerable number of spatial modes that can be guided within the limited core diameter, MMF-based endoscopes offer imaging resolutions down to the diffraction limit [20,21], which is ideal for the observation of synaptic dynamics.

For imaging, wavefront control is typically used to shape the light travelling through the MMF in order to compensate for perturbations in light propagation occurring inside the MMF [22–24]. For fluorescence imaging with point-scanning in the sample, the light propagation through the fiber must be characterized by performing a calibration procedure consisting of the acquisition of the transmission matrix (TM) that can describe the complex field transformation between two planes [25]. A wavefront-shaping device can then be used to shape the wavefronts coupled into the MMF to produce a focus at the distal end, which can be updated for digital scanning across the field-of-view [20]. However, the calibration, and thus the wavefront used for imaging, is highly sensitive to the relative position between the wavefront shaping device and the MMF. Any change in their relative positions will lead to a substantial degradation in the quality of the illumination focus and to an equivalent decrease in image quality. Repeated imaging with a MMF-based endoscope involves removing and reinserting a MMF implant into the system between each imaging session. This task is challenging since it is necessary to manually reposition the MMF into the optical system multiple times with sufficient (sub-micrometric) accuracy to preserve focus quality (peak intensity and size). This is complicated by the fact that it is not possible to repeat the standard TM measurement, as for *in vivo* chronic imaging the MMF will be implanted into an animal’s brain, and remain there for the duration of the experiment, which restricts the access to the distal end of the fiber rendering impossible the measurement of light output from the MMF.

Here we present a two-step method for repeated imaging using MMF. First, we designed a custom three-dimensional (3D) printed headplate, allowing precise reinsertion of the MMF implant into the optical system. This mechanical optimization step allows the creation of low-quality foci in the sample. Then, sensorless AO was implemented to correct for any remaining translational shift in the MMF position with respect to the wavefront shaping device in order to recover optimal focusing performance. Sensorless AO was based on the optimization of an image quality metric for different modes representing the optical aberrations present in the system. The performance of the proposed method was assessed by characterizing illumination foci and fluorescent bead images following multiple MMF removals and reinsertions. Moreover, we evaluated the effects of sensorless AO on imaging performance in living brain tissue.

## 2. Methods

### 2.1 Experimental system

The experimental system, generation of the TM and imaging procedure were previously described elsewhere [17] and a detailed experimental protocol was also made publicly available [26]. In short, a liquid-crystal spatial light modulator (LC-SLM, Meadowlark Optics, HSPDM512) was illuminated with light from a 488 nm continuous wave laser (CrystaLaser, DL488-020-S). The on-axis first-order diffraction beam, filtered in by an iris, carried the shaped wavefront and was coupled into a MMF after being relayed by 3 conjugated lenses (L1: 100 mm, L2: 50 mm, and L3: 8mm, focal lengths). The light exiting the distal end of the MMF was collected by an objective lens (L4, Olympus, PlanN 20, NA 0.4) and imaged (L5: 150 mm, focal length) onto a CCD camera (Basler pilot, piA640-210gm) for calibration or for characterizing the illumination. Using information from the TM and shaping the wavefront accordingly could form a focus at any lateral position at the distal calibration plane. Therefore, digital raster scanning was implemented to sequentially generate fluorescence at different focal locations. The fluorescence was then collected with the MMF, spectrally filtered before being detected by a photomultiplier tube (PMT).

### 2.2 Implant preparation, alignment, and calibration

Two different types of MMF were used: low numerical aperture (NA) MMF and medium NA MMF. Low NA MMF (Thorlabs, FG050UGA, core diameter 50 µm, NA 0.22, length 2 cm) glued inside a ferrule (Thorlabs, CF128-10) were used for experiments related to Figs. 2–5. Medium NA MMF (Doric lenses, custom MFC-ZF2.5 60/67/170, core diameter 60 µm, NA = 0.37, length 1.5 cm) were within a 170 µm glass/polyimide rigid coating and glued into a ferrule, and they were used for experiments related to Figs. 6 and 7. Ferrules were glued into the custom head plate and this assembly positioned into the imaging system using the magnets. The custom head plates were designed using AutoCAD software and 3D printed. They were made of MJF Nylon 12, a biocompatible material. 4 magnets were attached to both parts of the headplate (First4Magnets, F321-N35-50, 2 mm diameter and 1 mm height, N35 Neodymium). The weight of the headplate with a MMF and magnets was of 3.5 g. The headplate drawing is available upon request. For the initial alignment of the MMF implant into the optical system, the distal facet of the MMF was first imaged onto the calibration camera. The coupling lens (L3) was then translated axially to maximize coupling efficiency and laterally to be centered [26]. The CCD camera assembly was then translated axially by 50 µm away from the MMF distal facet for calibration. Calibrations were performed in the imaging medium [27] following the procedure described elsewhere [17,25,26].

### 2.3 Sensorless adaptive optics (AO)

Sensorless AO optimization was performed using 1) images of the illumination as captured by the CCD camera in the distal plane, or 2) the fluorescence signal detected by the proximal PMT. For both, illumination and fluorescence data, three aberration modes in the LC-SLM plane were tested in the following order: tip, tilt, and defocus. For each mode, 21 biases were sequentially superimposed on the wavefront dictated by the TM with an increment of 0.001 π rad for tip and tilt and of $4\times 10^{-6}\pi$ rad for defocus around zero bias (no aberrations). Next, the peak intensity was calculated and served as the quality metric, unless otherwise mentioned. A Gaussian fit was then performed on the quality metric to evaluate the optimal modal coefficient and the correction was immediately applied, before measuring the next mode. For the illumination case, the optimization was conducted by capturing images of a focus at a single, specified distal location. For the fluorescence case, the optimization was performed on the signal of a number of specified ROIs, in general between 1 and 6, taking advantage of the random-access capability of the system. Signals from the different ROIs were averaged together before Gaussian fitting. The time for sensing was less than 5 sec, when considering 6 ROIs.

### 2.4 Illumination characterization

Images (120×120 pixels) of the illumination foci were recorded with the distal CCD camera at the axial position at which the calibration was performed. We defined the upper-left corner of the image as pixel [1,1]. For evaluating different quality metrics, a focus was formed with varying amount of tip, tilt or defocus coefficient without displacement of the implant. Images were captured at 49 lateral positions arranged as a uniformly spaced grid (7×7) for each coefficient and mode. For assessing the performance of sensorless AO during sequential removals/reinsertions, the optimization was performed at pixel [45,60] (randomly selected) and illumination images acquired and analyzed when focusing at 49 lateral positions arranged as a uniformly spaced grid (7×7). To analyze the spatial variance in AO improvements across the FoV, the optimization was performed at pixel [45,60]. Illumination images were then acquired and analyzed for the 10 locations of the 7×7 grid described above located in the fourth octant. To analyze the spatial dependence of AO, sensorless optimization was performed at pixels [x,x], with x = {5 to 60 in increment of 5}. Illumination images were then acquired and analyzed when focusing at pixels {[45,60], [60,45], [75,60], [60,75]}.

### 2.5 Cultured hippocampal slices

Hippocampal slices (350 µm) were prepared from male Wistar rats (P7-P8). Brains were dissected and slices were cultured in culture media on Millicell CM membranes at 37 ∘C for 7–14 days prior to use.

### 2.6 Fluorescence imaging of beads

Beads samples were prepared using standard microscopy glass slides. Small amounts of Poly-D-Lysine were applied to the glass side and subsequently covered with diluted green fluorescent beads (FluoSpheres Carboxylate-Modified Microspheres, 1.0 µm, yellow-green fluorescent (505/515). For experiments related to Fig. 7, fluorescent beads (FluoSpheres Carboxylate-Modified Microspheres, 2.0 µm, yellow-green fluorescent (505/515) were immersed in agar, simulating the properties of fluorescently tagged neurons in mouse brains. The agar dilution was 0.5% to roughly match the mechanical properties of brain tissue. Agar pieces of 1.5 × 1.5 cm were glued onto microscope glass slides and covered with the top part of a small petri dish, serving as an ‘artificial skull’. Following the calibration procedure beads samples were mounted on a multi-axis stage below the MMF. For experiments in Fig. 5, glass slides covered with diluted beads (1 µm) was positioned approximately 100 µm below the MMF tip. While imaging, the sample was translated towards the MMF tip, until beads were in focus, evaluated by maximum signal. An image was acquired at this plane, serving as ‘Original’. Subsequently, the beads sample was moved downwards, giving enough space to manually remove the MMF implant. The MMF implant was fully removed from the optical system and rotated and flipped several times (Step 1). Then, the MMF implant was reinserted into the system via attaching the magnets on the implant to the magnets within the optical system (Step 2). Afterwards, the beads sample on the stage was moved up to the same plane that was used to capture the ‘Original’ image. Fine axial positioning in increments of 2 µm was performed, to determine the image plane that gives the highest signal. An image was taken at this plane, named ‘Reinsertion 1’ (Step 3). Sensorless optimization was performed choosing ROIs in the center of multiple beads. The image acquired was titled ‘Optimization 1’ (Step 4). Steps 1–4 were repeated three times. For experiments in Fig. 7, a small hole (2 × 2 mm) was drilled into the petri dish covering the agar with immersed beads (2 µm). Subsequently, the sample was transferred to the multi-axis stage and moved upwards until the full length of the fiber was inserted into the agar. While imaging, one fluorescent bead was positioned into focus and an image was acquired (‘Original’, Pre attachment). Then, the MMF implant was attached to the sample. First, super glue was used to attach the headplate to the petri dish. Next, dental cement was carefully applied to firmly fix the MMF implant onto the petri dish. Once the dental cement was dry, an image was taken to assess any shifts occurring during the attachment process (Post attachment). If the attachment was successful, the full construct of sample + MMF implant was removed from the optical system. Then, sample + attached MMF implant were reinserted and an image was taken (‘Reinsertion’). Sensorless optimization was performed choosing an ROI in the center of the bead that was originally positioned in focus.

### 2.7 Fluorescence imaging of hippocampal slices

Prior to imaging, brain slices were transferred to a recording chamber and individual hippocampal neurons in the CA3 region were loaded with Alexa 488 (2 mM) for 1 minute using whole cell patch-clamp technique. Slices were then transferred on a glass slide covered in Tyrode’s solution, and positioned in the MMF system, onto the multi-axis stage. Imaging was performed in physiological Tyrode’s solution to maintain slice health throughout the experiment. The hippocampal slice was positioned below the MMF, with the fiber tip directly above the CA3 region containing the neurons that have been filled with fluorescent dye. While imaging, neuronal processes such as thin dendrites were identified.

### 2.8 Image analysis

Peak intensity and lateral width analysis were performed on manually selected line profiles going through the center of illumination foci and beads for illumination and fluorescence data, respectively. The peak intensity was set as the maximum value of the line profile. The lateral width was evaluated as the FWHM of the profile and calculated using a MATLAB script [28]. The profile was first linearly rescaled by a factor of 11 before removing the background and normalizing it. Then, linear interpolation between the 2 points closest to 0.5 was performed on each side of the peak to find the 0.5-value positions. The difference between the 2 positions was the FWHM value. The signal-to-background ratio was calculated as the peak signal in the image divided by a background. The background was obtained by selecting a 20×20 pixels region where no structures were visible and measuring the average value.

## 3. Results

### 3.1 Mechanical and sensorless optimization

**Fig. 1.**
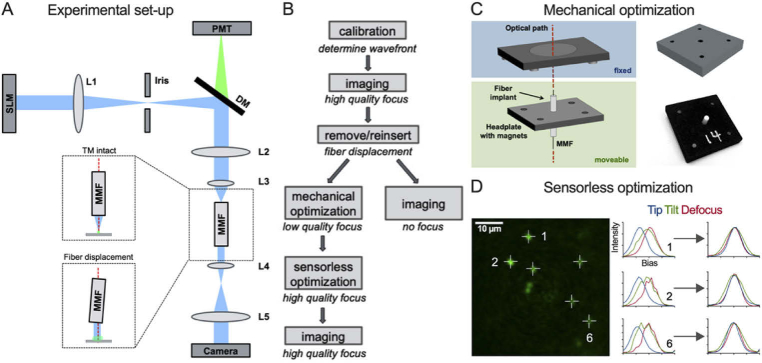
**Two-step approach for repeated imaging with a MMF.** (A) Schematic of the experimental set-up. Top inset shows focusing immediately after calibration and bottom inset shows loss of focusing capability if the MMF is displaced. (B) Flowchart of the two-step approach. (C) Schematic of the magnetic implant for mechanical optimization. (D) Illustration of sensorless AO.

The proximal MMF facet, the facet closest to the laser source, was located at a conjugated Fourier plane from the wavefront shaping device, in our case a liquid-crystal spatial light modulator (LC-SLM) (Fig. 1(A)). During the calibration procedure, the transmission matrix (TM) was measured to determine the wavefronts required to create high-quality foci in the sample for high-resolution imaging (Fig. 1(A)). Generally, the TM is considered to describe light propagation through complex media, such as the MMF itself [20,25]. However, light propagation between the LC-SLM and the proximal facet must also be considered in the TM. Indeed, the relative position of the MMF to the LC-SLM, and any optical element in-between, is critical for focus generation. Any displacement, even if the fiber remains straight and no bending occurs, will have a deleterious impact on the quality of the focus being formed in the sample. Removal and reinsertion of the MMF implant cause such positional changes between the wavefront shaping device and the MMF. These are typically so significant and the calibration so sensitive to any perturbation that no focus can be formed at the distal output of the MMF using standard approaches [17,18] (Fig. 1(A)).

To enable repeated imaging, a custom 3D printed headplate was designed to connect the MMF implant onto the animal and into the optical system. The MMF was attached in the center of the headplate and circled by 4 magnets (Fig. 1(B),(C)). The counterparts of those magnets were fixed into to the optical system, enabling repositioning after the MMF implant had been removed. This mechanical optimization procedure allowed for accurate reinsertions of the MMF implant sufficiently close to its original position such that a low-quality focus could be formed at the distal fiber facet (Fig. 1(C)). Further optimization of the focus quality was performed using sensorless AO. Sensorless AO is based on the optimization of an image quality metric for different modes representing the optical aberrations present in the system [5,29,30]. We considered the 3D shift between the MMF and the LC-SLM and therefore optimized for 3 modes at the LC-SLM plane: tip, tilt, and defocus. Each of these modes was sequentially corrected by applying different biases, finding the optima, and then superimposing the correction onto the shaped wavefront. Figure 1(D) exemplified this process where the fluorescence intensity (an image quality metric used in this work) is measured at 6 locations in the image. By applying different amount of tip, tilt and defocus successively, the fluorescence intensity is modulated with the peak of each mode being maximal at different biases. By correcting the wavefront by using the opposite biases, the optima of all modes are reached simultaneously.

**Fig. 2.**
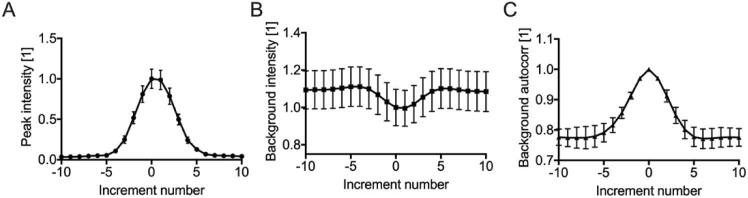
**Metrics for sensorless AO optimization**: (A) peak intensity, (B) background intensity, and (C) background autocorrelation. Each graph is normalized to the value at increment zero. An increment is equal to 0.001 π rad for tip and tilt and of $4\times 10^{-6}\pi$ rad for defocus around zero bias µm in wavefront correction applied to the LC-SLM.

When imaging the distal intensity distribution with a camera, several metrics can be evaluated. We quantified: signal intensity, background intensity and background autocorrelation. Figure 2 shows how the metrics changed when tilt was incrementally changed while focusing at different distal locations immediately after evaluating the TM (similar results were obtained for tip and defocus). The optimal tilt coefficient, here zero as there were no perturbations, could be correctly evaluated with all metrics. Critically, the SLM was sufficiently sensitive to impart wavefront modulations allowing for gradual changes in the metrics. The background intensity was minimal at the optimal tilt value, consistent with the fact that most of the intensity should reside in the focus when focusing optimal; however, the relative amplitude change was very limited (Fig. 2(B)). The background autocorrelation had a larger modulation but using this metric would not be compatible with endoscopic imaging without distal access (Fig. 2(C)). The peak signal intensity was maximal at the optimal tilt value, had the largest modulation and a minimal variance was observed across the different spatial locations (Fig. 2(A)). Moreover, considering the random-access capability of MMF-based imaging system, the peak intensity metric can be evaluated at a set of specified locations in the sample without the need to acquire a full image (Fig. 1(D)), thus providing an advantage in terms of correction speed. In short, the peak intensity appears to be the optimal metric for sensorless AO through a MMF.

### 3.2 Focus formation for repeated imaging using sensorless AO

**Fig. 3.**
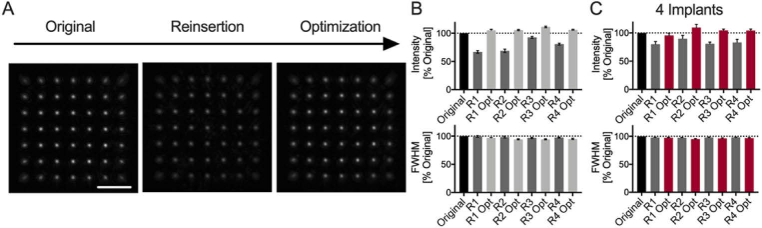
**Focusing light after multiple MMF reinsertion.** (A) maximum intensity projection of focus images at different lateral positions (left) immediately after calibration, (middle) after removal/reinsertion, and (right) after sensorless AO optimization. Scale bar: 20 µm. (B) Peak intensity and lateral width (FWHM) analysis of illumination foci across the FoV for the data shown in (A). (C) Peak intensity and lateral width analysis for 4 distinct implants following sequential removals/reinsertions and sensorless AO optimization. All data is normalized to the original values.

The quality of the illumination focus was assessed after sequential MMF implant removals and reinsertions. For this purpose, the illumination focus was imaged onto the distal camera without any sample being present. A calibration with a MMF-implant in the system was performed, then the MMF implant was completely removed and subsequently reinserted by attaching the magnets of the MMF implant plate to the magnets that were fixed in the system (Fig. 1(B)). Illumination foci were recorded just after the calibration (Original), after a number *i* of reinsertions R (R_i_) and after applying the sensorless optimization code (R_i,opt_; Fig. 3(A)). The intensity and full width half maximum (FWHM) of the illumination foci were quantified over a total of four reinsertions/optimizations. Intensities between 65% and 95% compared to the original intensity were measured following reinsertions (1 implant; Intensity [%]: R_1_: 67 ± 2, R_2_: 69 ± 3, R_3_: 92.6 ± 1.8, R_4_: 80.7 ± 1.7; Fig. 3(B)). Following sensorless optimization, intensities above that of the original focus quality were observed (1 implant; Intensity [%]: R_1,opt_: 105.1 ± 1.7, R_2,opt_: 105.6 ± 0.9, R_3,opt_: 111.2 ± 1.3, R_4,opt_: 106.2 ± 0.7; Fig. 3(B)). The FWHM decreased slightly for reinsertions and optimizations but stayed close to 100% compared to the original FWHM of the points (1 implant; FWHM [%]: R_1_: 99.6 ± 1.5, R_1,opt_: 96.8 ± 0.9, R_2_: 98.2 ± 1.5, R_2,opt_: 94.3 ± 0.9, R_3_: 96.8 ± 1.0, R_3,opt_: 94.3 ± 0.9, R_4_: 97.5 ± 0.8, R_4,opt_: 95.1 ± 1.1; Fig. 3(B)).

The previous measurements were performed with a single implant. To ensure that these results were consistently reproducible, independent of the MMF and headplate, these measurements were repeated with four different implants. Following reinsertions, intensity was on average over 80% for all reinsertions (4 implants; Intensity [%]: R_1_: 80 ± 5, R_2_: 90 ± 6, R_3_: 81 ± 3, R_4_: 83 ± 5; Fig. 3(C)) compared to the original, which could be improved to around 100% using sensorless optimization (4 implants; Intensity [%]: R_1,opt_: 96 ± 4, R_2,opt_: 110 ± 6, R_3,opt_: 105 ± 2, R_4,opt_: 104 ± 3; Fig. 3(C)). When assessing the FWHM, focus quality was similar compared to the original across all reinsertions (4 implants; FWHM [%]: R_1_: 97.9 ± 0.8, R_1,opt_: 97.5 ± 0.8, R_2_: 97.5 ± 0.7, R_2,opt_: 95.3 ± 0.7, R_3_: 98.0 ± 0.7, R_3,opt_: 96.4 ± 0.9, R_4_: 98.1 ± 0.7, R_4,opt_: 96.8 ± 0.9; Fig. 3(C)).

**Fig. 4.**
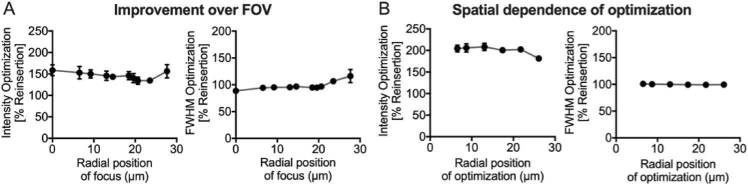
**Spatial dependence of sensorless AO in MMF.** (A) Peak intensity and lateral width analysis of illumination foci as a function of their radial position (distance from the core center) after performing sensorless AO optimization near the fiber center. (B) Peak intensity and lateral width analysis of illumination foci across the FoV as a function of the radial position at which sensorless AO was performed. All data is normalized to the pre-AO values.

Uniform focusing performance across the field-of-view (FoV) ensures uniform sampling of objects and is important as neuronal structures will typically span across the entire FoV [31]. We assessed the impact of sensorless AO at different locations in the FoV after optimizing for a focus near the center of the FoV. The FWHM and peak intensity of illumination foci were quantified as a function of their distance from the center of the FoV and constant improvements were measured across the FoV (Fig. 4(A)). Sensorless optimization was performed at different radial positions and the changes in focusing characterized across the FoV. No spatial dependence was observed for the improvement in intensity or lateral width, indicating that sensorless AO following fiber reinsertion is not dependent on the position of the optimization point (Fig. 4(B)).

In summary, the magnetic headplate was sufficient to partially preserve focusing capability across multiple MMF implants following several reinsertions. Combined with sensorless AO, focus characteristics equivalent to pre-removal (original) were achieved by correcting only for tip, tilt, and defocus.

### 3.3 Repeated fluorescence imaging of beads through a MMF

**Fig. 5.**
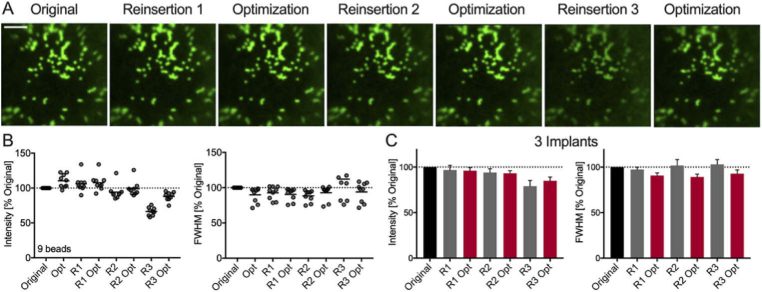
**Repeated imaging of 1 µm fluorescent beads.** (A) Images of the same sample acquired from left to right after performing removal/reinsertion and sensorless AO optimization. Scale bar: 15 µm. (B) Peak intensity and lateral width analysis for the bead imaging data shown in (A). (C) Peak intensity and lateral width analysis for 4 distinct implants following sequential removals/reinsertions and sensorless AO optimization. All data is normalized to the original values.

For chronic *in vivo* imaging, the MMF must be implanted in the brain and the headplate attached to the skull. Hence, the distal camera cannot be used for capturing the illumination as the sensorless optimization signal. Instead, the optimization should be based on the signal of interest for imaging. Neurons are typically fluorescently labeled via viral injections or endogenously express a fluorescent protein [32,33]. As such the proximally detected fluorescence was employed for sensorless optimization. To assess the performance of the proposed repeated imaging methods, 1 µm fluorescent beads were chosen, as they are similar in size to synaptic terminals such as boutons and spines (<2 µm). Beads were imaged immediately after calibration, after removing and reinserting the implant, and after performing AO (Fig. 5(A)). Even after three reinsertions, image quality remained high thanks to the custom designed headplate, allowing for precise reinsertion, as illustrated for a single implant in Fig. 5(A),(B) (9 beads; 1 implant; Intensity [%]: Original: 99 ± 4, R_1_: 106 ± 4, R_1,opt_: 108 ± 4, R_2_: 94.1 ± 0.7, R_2,opt_: 95.3 ± 0.7, R_3_: 66 ± 2, R_3,opt_: 88 ± 2; FWHM [%]: Original: 90 ± 4 R_1_: 93 ± 3, R_1,opt_: 91 ± 3, R_2_: 88 ± 3, R_2,opt_: 93 ± 3, R_3_: 112 ± 11, R_3,opt_: 94 ± 5). Importantly, when reinsertions were mechanically less successful, sensorless optimization provided greater relative improvements and was able to bring the peak intensity near the original values (as seen from R_3_ to R_3,opt_ in Fig. 5(A),(B)). Overall, peak intensity did not drop below 80% following MMF removal and reinsertion compared to the original image and FWHM stayed close to 100% (25 beads; 3 implants; Intensity [%]: R_1_: 97 ± 5, R_1_,opt: 96 ± 3, R_2_: 94 ± 4, R_2,opt_: 93 ± 3, R_3_: 79 ± 6, R_3,opt_: 85 ± 4; FWHM [%]: R_1_: 97 ± 3, R_1,opt_: 91 ± 3, R_2_: 102 ± 7, R_2,opt_: 89 ± 3, R_3_: 103 ± 5, R_3,opt_: 93 ± 4; Fig. 5(C)). These results demonstrate that repeated high-resolution fluorescence imaging is feasible with the proposed chronic MMF implant when combined with AO.

### 3.4 Sensorless AO improves imaging of live neurons

While fluorescent beads are similar in size compared to synaptic structures, their brightness is much larger than that of living fluorescent brain structures. Moreover, the beads samples were inherently two-dimensional (2D) and had little background, while brain tissue will contain 3D fluorescent structures and have a lower signal-to-background ratio due to out-of-focus signal. Additionally, the insertion of the fiber into tissue may cause bending and translation of the MMF as a result of physical forces exerted by the tissue. To limit the effects of bending, custom made fibers were coated with a rigid tubing [34]. Thereby, the MMF was subjected to less deformation when inserted into tissue. However, translation and some degree of fiber bending may be inevitable.

**Fig. 6.**
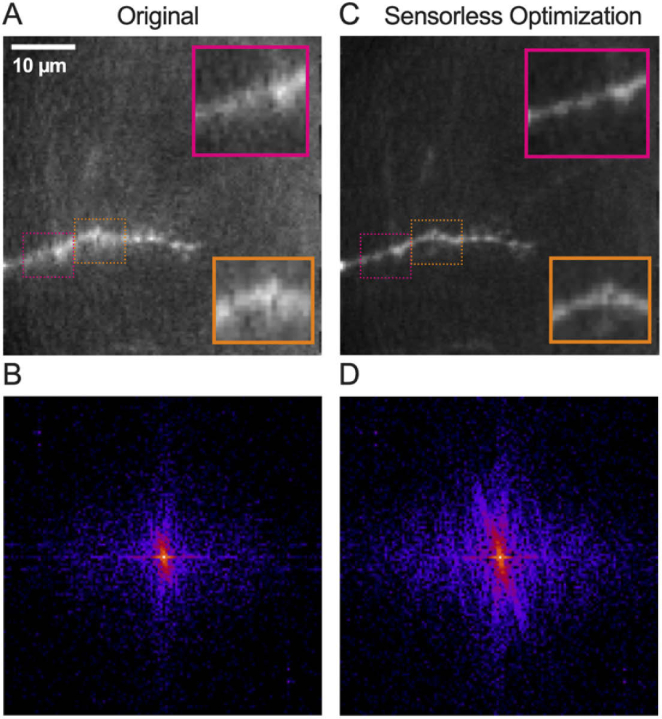
**Imaging of live neurons through an MMF in slices with sensorless AO.** (A,C) Images of a dendrite labeled with Alexa Fluor 488, acquired (A) before and (C) after sensorless AO optimization. Scale bar: 10 µm. Inset width: 10 µm. Images were normalized to their peak intensity. (B,D) Power frequency spectra of the images shown in (A,C), respectively.

To evaluate whether the remaining effects of these static aberrations occurring during insertion can be corrected by sensorless AO, live brain tissue was imaged (Fig. 6). Only translational modes (tip, tilt and defocus) were evaluated to ensure that the corrections would apply across the FoV. CA3 pyramidal neurons were filled via patch-clamp technique in organotypic hippocampal slices with a fluorescent dye (Alexa 488). Subsequently, slices were transferred to the MMF-based system and imaged immediately after calibration. Neuronal processes, including dendrites could be identified as bright structures (Fig. 6(A),(C)). The original signal-to-background ratio was calculated to be of 2.7 in the displayed image (Fig. 6(A)). Following sensorless AO, a greater level of detail could be visualized with small protrusions known as postsynaptic terminals or spines becoming more visible (Fig. 6(C)). A substantial relative decrease in background was achieved with the signal-to-background ratio increasing by a factor of 2.3. The improvement provided by correcting for tip, tilt, and defocus is further evidenced by the increased spatial frequency bandwidth as shown by the Fourier transforms of the images (Fig. 6(B),(D)). These results indicate that the sensorless optimization can indeed be used in living brain tissue to improve image quality.

### 3.5 Repeated imaging of 3D samples

**Fig. 7.**
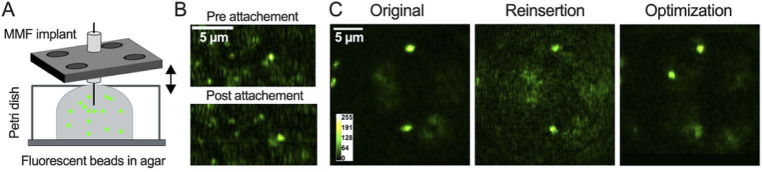
**Repeated imaging in a 3D sample.** (A) Schematic representation of the sample consisting of 1–2 µm green fluorescent beads imbedded in agar. (B) Imaging (top) before and (bottom) after affixing the implant onto the petri dish with glue and dental cement. (C) Images of a sample as shown in (A) (left) after attachment of the implant to the sample, (middle) after removal and reinsertion, and (right) after sensorless AO optimization. Image width: 54 µm.

To perform chronic imaging in living animals, the implant must be attached onto the animal’s skull, after the fiber is positioned at the region of interest. In other words, after imaging the desired neuronal structure, the implant must be carefully fixed in place without disrupting the calibration. To test whether this was possible, 3D fluorescent beads were immersed in agar, thus simulating the properties of fluorescently tagged neurons in mouse brains (Fig. 7(A)). The agar dilution was chosen to roughly match the mechanical properties of brain tissue. Afterwards, a petri dish was attached to cover the bead-agar mixture and provide a solid substrate for attaching the implant. Then, a small hole was drilled into the petri dish, such as for craniotomy, and the MMF was inserted into the agar. After having identified a region of interest, with fluorescent beads in focus (Fig. 7(B)), the implant was attached to the petri dish with superglue and dental cement while carefully avoiding any disruptions of the system. Images were recorded before and after the implant was attached to the sample, to evaluate potential shifts or disruptions and they show that we can manually attach the implant to the sample without causing any major distortions to the fiber position (Fig. 7(B)).

In another experiment, the capacity for repeated imaging was evaluated after attachment of the implant to the sample. The sample was first imaged after calibration and implant insertion and attachment (Fig. 7(C), left). Next, MMF implant + sample were removed together as a unit, from the optical system and subsequently reinserted. The same beads were visible and in focus after magnet-based reinsertion (Fig. 7(C), middle). Sensorless AO optimization was then performed on the top-most bead and a substantial reduction in the background was observed (Fig. 7(C), right). An axial shift was also noticeable and was caused by the top-most bead initially not being in focus and being the only optimization point. Indeed, defocus can propagate through the fiber [35] and our sensorless implementation will thus automatically bring objects of interest into focus. Alternatively, the defocus can be manually adjusted to image the same axial plane using morphological similarity or by selecting multiple objects to maintain the average axial location for sensorless AO (e.g.: Fig. 6). To ensure that the same axial plane is imaged between imaging sessions, it is recommended to perform an AO optimization at a given set of locations immediately after calibration and keep these locations for future optimization. In conclusion, we can successfully attach the implant with glue and dental cement while preserving image quality and enabling repeated imaging with a MMF-based endoscope and sensorless AO.

## 4. Discussion

We have presented a method for repeated high-resolution imaging with a MMF-based micro-endoscope by precise manual reinsertion of the chronic MMF implant using magnets, followed by further positional optimization using sensorless AO. MMF-based endo-microscopes are particularly well suited for AO as the experimental system already includes a wavefront shaping device, here a LC-SLM. Moreover, no modifications of the instrument were required for a sensorless implementation, as no dedicated wavefront sensors were needed. Indeed, the optimization was conducted using the same photodetector as the one used for imaging. Our data showed that even after careful repositioning of the fiber implant, the quality of the focus could be enhanced with sensorless AO. It should be reiterated that AO corrected the positional shift (misalignment) between the optical system and the proximal facet of the MMF. As such, physical fiber parameters influencing light propagating inside the fiber, including the number of modes, should not have any effect of the ability of AO as it was implemented here to recover optimal focusing performance.

Biological samples often introduce optical aberration due to the heterogeneity in their refractive index distribution or mismatch at interfaces [5,29,30]. These optical aberrations deteriorate image quality and AO is generally employed to correct for such specimen–induced aberrations and thus provides increased fluorescence signal contrast, and spatial resolution [5,29,30]. We have previously shown that, for MMF endo-microscopy, considering the refractive index of the biological object by performing the calibration in an index-matched solution led to improved focusing ability in brain tissue [27]. For repeated imaging with a MMF, system aberrations are predominant because the system is highly sensitive to any alteration in the relative position between the MMF and the LC-SLM. Hence, we introduced AO to correct for aberrations in the system’s illumination path.

In this study, we corrected translational changes in the position of the fiber proximal facet, which translate into tip, tilt, and defocus in the LC-SLM located in a conjugated Fourier plane. The proximal fiber facet can also be tilted or rotated during repositioning, respectively corresponding to translation and rotation in the LC-SLM plane. Correction for the latter parameters was not necessary, as the custom headplate allowed us to reposition the implant precisely enough – with minimal fiber tilt and rotation– to ensure high image quality. Our data support this idea because in all cases imaging performances equivalent or close to the unperturbed system were recovered. Nevertheless, it could be beneficial to introduce further correction modes and even other correction basis for other applications. For instance, sensorless AO could be combined with other strategies to measure and optimize the altered TM after static deformation (as suggested per Fig. 7). We anticipate that our approach will be compatible with the use of distal reflectors [36,37], metasurfaces [38], single mode channels [39], or ultraprecise parabolic-index fibers [40]. The design of the AO optimization scheme in more complex cases could also take advantage of the memory effect [41]. In any cases, the use of a rigid tubing, as was used in this work, is recommended to minimize any bending or twisting of the fiber during its insertion in biological tissue. We expect such rigid tubing to be sufficient to enable reaching any brain regions in mice with minimal deformation.

Our results showed that mechanical repositioning of the MMF implant alone was sufficient for repeated imaging of fluorescent beads without loss of spatial resolution; only a decrease in signal intensity was observed and recoverable with sensorless optimization. In these 2D bead samples, the absence of out-of-focus objects contributed to the signal-to-background ratio and the image quality remaining high in most cases. However, similar amounts of MMF misalignment after manual repositioning resulted in much larger backgrounds for 3D samples (Figs. 6 and 7) due to out-of-focus fluorescence. In such cases, sensorless AO optimization substantially increased the information content of images. The implementation of AO would also be beneficial for multiphoton fluorescence imaging through MMF, even as limited background would be generated, because the signal depends nonlinearly on the illumination power in the focus [34,42]. We reiterate that there was no spatial dependence as to where the optimization was performed. Moreover, the optimization stayed relatively constant across the fiber facet, enabling enhanced image quality across the field-of-view.

The use of a LC-SLM was not optimal for deployment of the proposed method in living tissue. Indeed, the relatively slow update rate of the LC-SLM led to a long pixel dwell time. Such pixel dwell time might have deleterious effects on cellular health when combined with the sensorless measurements, involving different modes and multiple biases for each, at the a few unique spatial locations within a cell. The use of a digital micromirror device for wavefront shaping, which has been demonstrated in the living brain, would be preferable due to a much faster update rate [18].

## 5. Conclusion

This study introduced a method for repeated imaging using MMF. Manual repositioning of MMF presents unique obstacles, as a change in relative positioning with respect to the wavefront shaping device invalidates the original calibration, necessary for foci formation and imaging. We tackled this problem using custom 3D printed headplates enabling precise reinsertion of the MMF implant, thus preserving low-quality focusing that can subsequently be transformed into high-quality focusing using sensorless AO. We believe that our methodology constitutes an important step towards chronic high-resolution imaging of deep brain structures in living animals using the minimally invasive MMF technology. Such repeated *in vivo* imaging demonstration will be the goal of future work.

## Data Availability

Data underlying the results presented in this paper are not publicly available at this time but may be obtained from the authors upon request.
